# Effect of Erbium-yttrium, scandium, gallium and garnet (Er-YSGG) laser on the bond strength of lithium disilicate ceramics

**DOI:** 10.12669/pjms.341.13916

**Published:** 2018

**Authors:** Mohammed Qasim Al Rifaiy

**Affiliations:** Mohammed Q. Al Rifaiy, Department of Prosthetic Dental Sciences, College of Dentistry, King Saud University, Riyadh 11545, P.O. Box: 60169, Saudi Arabia

**Keywords:** Bond strength, Laser, Lithium disilicate ceramic, Resin composite, Surface treatment

## Abstract

**Objectives:**

To assess the bond strength of LD ceramics with resin composite material and surface conditioning using Er: YSGG laser and HF acid.

**Methods:**

Thirty LD ceramic (Emax, Ivoclar vivadent) discs were prepared using hot pressing technique and treated with hydroflouric acid (Group-1-HF acid) (9%) (n=10) and Er- yttrium, scandium, gallium and garnet laser (Group-2-ER-YSGG laser) (Waterlase iPlus, 10 Hz and power of 0.5 W, pulse duration of 230 μs) (n=10). Ten specimens were left untreated to be included as controls (Group-3-Control). All the specimens were treated with Adper Single Bond adhesive (3MESPE, St. Paul, MN, USA). Multicore buildups (3mmx3mm) were performed using a rubber mold on the ceramic surfaces and cured using LED light-curing unit for 140 sec. All specimens were tested using shear bond test and failure modes were assessed with a stereomicroscope and scanning electron microscope. Data was analysed using ANOVA and Tukey Kramer multiple comparisons test.

**Results:**

The maximum and minimum shear bond strength values were achieved in HF Acid specimens (Group-1) (28.15±4.72 MPa) and control specimens (13.47± 3.14 MPa) respectively. Specimens treated with HF acid showed significantly higher bond strength in comparison to laser treated and control specimens (p<0.01). Laser treated specimens had significantly higher bond strength as compared to controls (p<0.01).

**Conclusions:**

Hydrofluoric (HF) acid treatment showed significantly better outcomes than YSGG laser surface treatment.

## INTRODUCTION

All ceramic restorations are increasingly used for oral rehabilitation due to improved esthetics.^1^ Lithium disilicates (LD) are dental ceramics offering different levels of translucency and ability to adhesively bond to tooth structure.^2^,^3^ However the strength of LD ceramics is not optimal, therefore are not indicated for posterior FPDs.

An important feature of LD ceramics is bonding to tooth structure resulting in a monobloc of tooth and ceramic restoration. However this adhesive bond includes multiple clinical and laboratory steps which have a critical role in clinical success of LD ceramics. The surface conditioning of LD ceramics is conventionally performed by hydrofluoric (HF) acid (5% to 9%).^4^,^5^ HF acid is a caustic and hazardous substance and cannot be used intra-orally. In addition, application of HF acid for longer durations and with high concentrations, have shown a reduction in the mechanical strength of LD ceramics.^6^-^9^

Recently, lasers have been introduced in dentistry with multiple applications including, surgery, removal of soft tissue pigmentation, tooth whitening and periodontal therapy. ER-YSGG laser was introduced in operative dentistry for the removal of dental caries, conditioning of tooth surfaces and conditioning of enamel and dentin.^10^-^12^ It is suggested that the ablative effect of ER-YSGG laser can result in temperature rise and morphological changes in tooth structure. In addition, ER-YSGG lasers have been experimentally used in the surface conditioning of ceramics, including zirconia.^10^,^13^ HF acid cannot be used intra-orally and has the potential to be harmfull to oral tissues, and the effective use of lasers can be a safe alternate for the conditioning of LD ceramics.

Therefore we hypothesize that the use of ER-YSGG laser in the surface coditioning of LD ceramics would be equally effective to the use of HF acid. The aim of this study was to assess the bond strength of LD ceramics with resin composite material with the surface conditioning using Er-YSGG laser and HF acid.

## METHODS

Thirty (3mm x 6mm) LD ceramic (Emax, Ivoclar vivadent) discs were prepared using hot pressing technique according to manufacturers instructions. The surface of the discs after devesting were cleaned using ultrasonic cleaner and the specimens stored in distilled water for 24 hours. Ten specimens (Group-1) were treated with hydroflouric acid(Group-1-HF acid) (9%), applied using disposable brush covering the complete surface of the disc for 90 seconds (sec). The HF acid was cleaned and surface dryed for two minutes (mins). Another 10 specimens (Group-2-ER-YSGG laser) were treated with ER-YSGG laser [Waterlase iPlus, 10 Hz and power of 0.5 W, pulse duration of 230 μs (very short pulse)] in a non-contact mode. All treated specimens were cleansed using distilled water in an ultrasonic bath for five minutes. 10 specimens were left untreated (without any surface conditioning) (Group-3-Control).

For bonding procedures, all the specimens were rinsed for 10 sec (distilled water) and air dried for five seconds. A single layer of ceramic primer in the form of silane (RelyX TM Ceramic Primer) was applied to all samples. Following this Adper Single Bond adhesive (3MESPE, St. Paul, MN, USA) was applied and cured for 10 sec with LED light curing unit (800 mW/cm2) (DEMI, Kerr, Ca, USA). Multicore buildups (3mmx3mm) were performed using a rubber mold on the ceramic surfaces and cured using LED light-curing unit 140 sec. Specimens in each Group (1, 2 and 3) were tested using shear bond test with Instron universal testing machine, at a standard load and 0.5mm crosshead speed. Authors were blinded from the specimen groups at bond strength testing.

Failure mode was assessed for all specimens in each group with a stereomicroscope (Nikon C-DS, Tokyo, Japan) at 50X magnification and scanning electron microscope (SEM, XL 30CP, Phillips, MA, USA). Specimens were mounted and alcohol wiped, sputter coated with gold for 180 seconds at 40mA, creating a 30nm thick layer. Samples were examined under different standard magnifications of SEM operated at 20KV using secondary electron detection. Failure modes were divided into, Adhesive failure at the tooth/composite interface, Cohesive failure in resin, cohesive failure in tooth and mixed failures. All data passed normality which was tested using the Kolmogorov-Smirnov test. Data was analysed using ANOVA and Tukey Kramer multiple comparisons test.

**Fig. 1:**
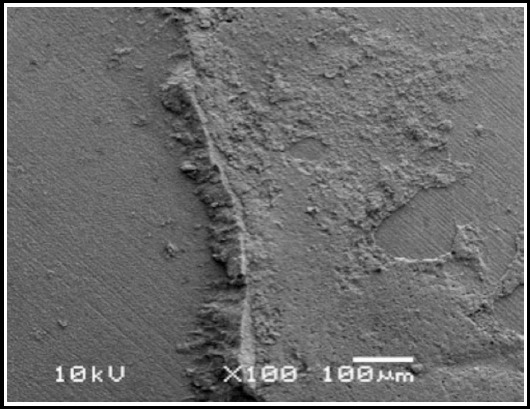
SEM image of adhesive failure in HF acid treated specimen.

**Fig. 2:**
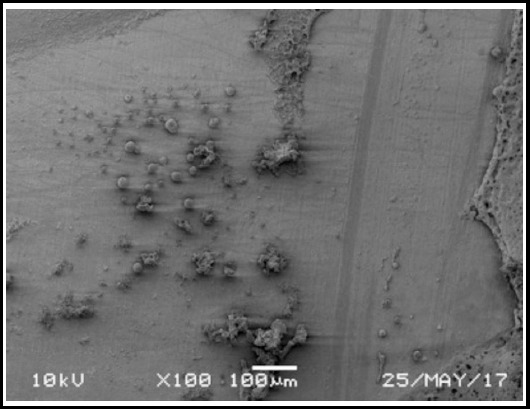
SEM image of mixed failure in Er-YSGG laser treated specimen.

## RESULTS

The study assessed the influence of laser (Er-YSGG) surface treatment in comparison to HF acid and control specimens on the bond strength of lithium disilicate ceramics.

All data passed the normality test, assessed using Kolmogorov-Smirnov test. The maximum shear bond strength values were achieved in HF Acid samples (Group-1) (28.15±4.72 MPa). The minimum shear bond strength values were shown by control specimens (Group-3) (13.47± 3.14 MPa) (Table-I). The mean shear bond strength for laser (YSGG) specimens was 22.83 ± 5.07 MPa. Specimens treated with HF acid showed significantly higher bond strength in comparison to laser treated and control specimens (p<0.01). Laser treated specimens had significantly higher bond strength as compared to controls (p<0.01) (Table-I). Specimens in Groups (1, 2 and 3) showed 70%, 50% and 100% of adhesive failures. Specimens in Groups-1 and 2 had 30% and 50% of mixed failures (Table-II).

**Table-I:** Means and standard deviations of the observed bond strengths among study groups.

Study Groups	Mean	SD	P-value
HF Acid (Gp 1)	28.15a	4.72	< 0.001*
Laser (Gp 2)	22.83b	5.07	
Control (Gp 3)	13.47c	3.14	

HF: hydroflouric, Laser: ER-YSGG, Control: no ceramic conditioning.

Means compared using ANOVA.

* Statistical significance. Groups marked with disimilar letters are signifcantly different (Tukey Kramer Multiple comparisons test).

**Table-II:** Distribution of failure modes among study groups.

Study Groups	Adhesive (n)	Mixed (n)	Cohesive (0)
HF Acid (Gp 1)	70% (7)	30% (3)	0 % (0)
Laser (Gp 2)	50% (8)	50% (2)	0 % (0)
Control (Gp 3)	100% (10)	0 % (0)	0 % (0)

## DISCUSSION

The present study was based on the hypothesis that the use of ER-YSGG laser in the surface coditioning of LD ceramics would be equally effective to the use of HF acid in their adhesicve bonding. The investigation showed that shear bond strength for YSGG laser treated LD ceramic specimens were significantly lower as compared to HF acid treated specimens. Therefore the hypothesis was rejected.

Lithium disilicate ceramics are extensively used in dentistry and are even advocated for anterior three-unit fixed partial dentures. Along with its high crystalline phase, it has the ability to adhesively bond to tooth structure critically based on successful surface treatment.^14^,^15^ Hydroflouric acid is considered a standard in the treatment of LD ceramic restorations for surface treatment, for effective adhesive bonding of these restorations. Many studies have shown the efficacy of HF acid in the adhesive bonding of different forms of dental glass ceramics.^16^ In addition, application of silane coupling agents provide chemical bond between the silica of ceramics and molecules within silane to improve the wetting of the etched ceramic and to promote resin penetration.^17^,^18^ In the present study resin composites were used as substrate for bonding, as the aim was to assess the ceramic surface treatment of bonding interface and to minimize the variables like tooth enamel and dentine quantity and quality and number of dentine tubular openings.^19^

The present study showed that the use of Er-YSGG laser showed lower bond strength of ceramics to resin as compared to HF acid treatment. Lasers in dentistry have developed for the last decade and different forms of lasers (including Diode, CO_2_, Er-YAG, ND-YAG lasers) have been employed for application of soft and hard tissue cutting, disinfection and fluid activation.^20^,^21^ Recently, studies investigating the influence of lasers in treating ceramic surface for effective bonding have been reported.^22^,^23^ However different laser types and protocols have resulted in a controversy regarding the efficacy of lasers in ceramic surface treatment.^24^ In a study by Gokce et al., LD ceramic surface was treated with Er-YAG laser at a wavelength 2940 nm and 300 to 900mJ power.^24^ Specimens treated with 600 and 900mJ power showed significantly lower bond strengths as compared to HF specimens. However specimens exposed to laser at 300mJ power showed comparable bond strength values to HF acid treated samples. In the present study YSGG laser operated at 500mJ/sec, however the outcomes showed lower bond strength of ceramics as compared to HF acid treatment. The laser was operated at 500mJ as YSGG lasers are known to deliver low amounts of energy as compared to Er-YAG laser as reported in the study by Gokce et al.^24^ This could be attributed to the fact that short burst of temperature rise on the ceramic surface cause thermal vaporization of the substrate. This results in a heat damaged ceramic surface layer, which during shear bond testing shows cohesive failure from the subsurface ceramic.^25^ The surface layer however bonds strongly with the resin composite. Therefore it is suggested that YSGG laser at lower power would be more beneficial as compared to higher power. Further studies assessing the effect of YSGG laser surface treatment of LD ceramics on their bond strength with different protocols are recommended in this regard.

Interestingly, the failure modes in the present study for the HF acid treated specimens predominantly were Adhesive (70%). By contrast 50% of the laser treated specimens showed mixed failures with adhesive interface and ceramic surface layers. This is attributed to the thermal ablation of the ceramic surface in some areas of the specimens, weakening the surface layer.^25^ The lower bond strengths and higher mixed failures for YSGG laser treatment also reflects the irregular and insufficient ceramic surface patterns, inhibiting resin and silane penetration in the ceramic.

A possible limitation was the number of exposures of laser on ceramic surface, which in this study was only one. Incresing the numbr of exposures may improve the bond strength values. The effect of ER-YSGG laser on the surface of tooth is known to be influenced by the laser parameters (frequency, wavelength and power). However laser application in the present study included 10 Hz and power of 0.5 W. Application of laser with incresed frequency and power could have resulted in a different bond strength outcome. Therefore, further studies assesing different number and duration of laser exposure with varying parameters are recommended to assess their influence on LD ceramic bond strength.

Clinically speaking, lasers do show potential for the surface treatment of LD ceramics for improvement in the resin bond strength, however further investigations with different combinations of laser protocols (wavelength and power) are recommended.

## CONCLUSION

Within the limitations of the study, YSGG Laser treatment of LD ceramics showed significantly better bond strength as compared to untreated sepcimens. HF acid treatment showed significantly better outcomes than laser surface treatment. Further investigations into the effect of different laser protocols are recommended.
